# Complete plastome sequence of *Atalantia kwangtungensis* (Rutaceae): an endemic “near threatened” shrub in South China

**DOI:** 10.1080/23802359.2018.1483764

**Published:** 2018-07-03

**Authors:** Zhi-Xin Zhu, Jian-Hua Wang, Xun-Zhe Sun, Kun-Kun Zhao, Hua-Feng Wang

**Affiliations:** Hainan Key Laboratory for Sustainable Utilization of Tropical Bioresources, Institute of Tropical Agriculture and Forestry, Hainan University, Haikou, PR China

**Keywords:** *Atalantia kwangtungensis*, illumina sequencing, plastome, Rutaceae, phylogenetic analysis, Sapindales

## Abstract

*Atalantia kwangtungensis* (Rutaceae) is a small shrub (1–2 m tall) distributed in moist and shady places throughout evergreen broad-leaved forests having altitudes that range from 100 to 400 m in West Guangdong, Southeast Guangxi, Hainan province of China. It has been ranked as a near threatened (NT) species in China. Here, we report and characterize the complete plastid genome sequence of *A. kwangtungensis* in an effort to provide genomic resources useful for its conservation. The complete plastome is 160,248 bp in length and contains the typical structure and gene content of angiosperm plastomes, including two inverted repeat (IR) regions of 27,151 bp, a large single-copy (LSC) region of 87,483 bp and a small single-copy (SSC) region of 18,463 bp. The plastome contains 110 genes, consisting of 77 unique protein-coding genes (ycf1 is a pseudogene), 29 unique tRNA genes, and 4 unique rRNA genes. The overall A/T content in the plastome of *A. kwangtungensis* is 61.6%. Phylogenetic analyses were performed using the entire plastome, including spacers, introns, etc., and we determined that *A. kwangtungensis* and *Merrillia caloxylon* were closely related. The complete plastome sequence of *A. kwangtungensis* will provide a useful resource for the conservation genetics of this species as well as for the phylogenetic studies in Sapindales.

*Atalantia kwangtungensis* Merr. (Rutaceae) is a small shrub (1–2 m tall) distributed in moist and shady places throughout evergreen broad-leaved forests having altitudes that range from 100 to 400 m in West Guangdong, Southeast Guangxi, Hainan province of China (Zhang et al. 2008). It has been ranked as a near threatened (NT) species in China (Ministry of Environmental Protection of the Peoples-Republic of China and Chinese Academy of Sciences 2013). Consequently, genetic, and genomic information is urgently needed in order to promote its conservation of *A. kwangtungensis*. Here, we report and characterize the complete plastome of *A. kwangtungensis* (GenBank accession number: MH329190, this study) based on Illumina paired-end sequencing data.

In this study, *A. kwangtungensis* was sampled from Dahuajiao of Wanning city Nature Reserve in Hainan province of China (110.53°E, 18.79°N). A voucher specimen (H. F. Wang et al. B237) was deposited in the herbarium of the Institute of Tropical Agriculture and Forestry (HUTB), Hainan University, Haikou, China.

The modified cetyltrimethylammonium bromide (CTAB) protocol of Doyle and Doyle (1987) was used to extract genomic DNA from dry leave tissues. The genomic DNA of each sample was quantified and analysed with Agilent 2100 BioAnalyzer. Samples yield at least 0.8 μg DNA was selected for subsequent libraries construction and de novo sequencing. Genomic DNA of selected samples was used to build the paired-end libraries with 200–400 bp insert size. Libraries were sequenced using BGISEQ-500 platform at BGI Shenzhen, China and produced about 8 Gb high quality per sample with 100 bp paired-end reads. Raw reads were trimmed using SOAPfilter_version 2.2 (Shenzhen, China) with the following criteria (1) reads with >10% base of N; (2) reads with >40% of low quality (value < =10); (3) reads contaminated by adaptor and produced by PCR duplication. Around 6 Gb clean data for each sample were used to perform the assembling of chloroplast genome against the plastome of *Zanthoxylum schinifolium* (Genbank Accession number: KT321318.1) using MITObim version 1.8 (Oslo, Norway) (Hahn et al. 2013).

Plastomes were annotated using Geneious R8.0.2 (Biomatters Ltd., Auckland, New Zealand) against the plastome of *Z. schinifolium* (Genbank Accession number: KT321318.1). The annotation was corrected with DOGMA (Wyman et al. 2004). A circular plastome map was generated using OGDRAW (http://ogdraw.mpimp-golm.mpg.de/) (Lohse et al. 2013).

The plastome of *A. kwangtungensis* was found to possess a total length 160,248 bp with the typical quadripartite structure of angiosperms, containing two inverted repeats (IRs) of 27,151 bp separated by a large single-copy (LSC) region and a small single-copy (SSC) region of 87,483 and 18,463 bp, respectively. The plastome was found to contain 110 genes, including 77 protein-coding genes (seven of which are duplicated in the IR), four ribosomal RNA genes, and 29 tRNA genes (six of which are duplicated in the IR). Among these genes, ycf1 (translation from 134185 to 128691) is a pseudogene, 14 genes (*trnA-UGC, trnI-GAU, trnK-UUU, trnL-UAA, trnV-UAC*, *atpF, ndhA, ndhB, petB, petD, rpoC1, rpl2, rpl16,* and *rps16*) harbored a single intron and three genes (*ycf3, clp*P, and *rps12*) had two introns. The gene *rps12* has trans-splicing. The overall A/T content of the plastome was 61.6%, while the corresponding values of the LSC, SSC, and IR regions were 63.2, 66.7, and 57.1%, respectively.

We used RAxML (Stamatakis 2006) with 1000 bootstraps under the GTRGAMMAI substitution model to reconstruct a maximum likelihood (ML) phylogeny of nine published complete plastomes of Rutaceae, using *Leitneria floridana* (*Simaroubaceae,* Sapindales) as an outgroup. The phylogenetic analysis indicated that *A. kwangtungensis* and *Merrillia caloxylon* are closely related and all members of Rutaceae were clustered with a high bootstrap support (BS) value (Figure 1). The *A. kwangtungensis* plastome reported here will provide a useful resource for the development of medicinal and edible value as well as for phylogenetic studies of Sapindales.

**Figure 1.**
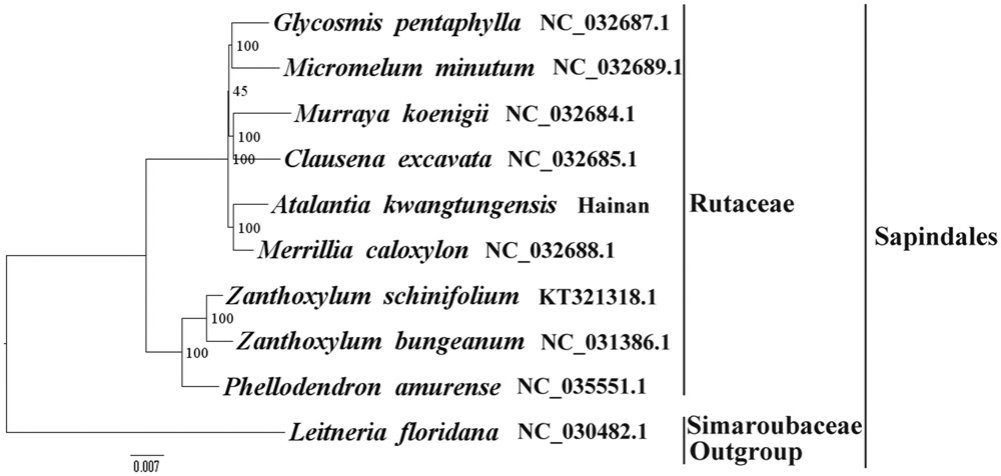
The best ML phylogeny recovered from 10 complete plastome sequences by RAxML. Accession numbers: Atalantia kwangtungensis (this study, GenBank accession number: MH329190), Merrillia caloxylon NC_032688.1, Murraya koenigii NC_032684.1, Clausena excavata NC_032685.1, Micromelum minutum NC_032689.1, Glycosmis pentaphylla NC_032687.1, Zanthoxylum bungeanum NC_031386.1, Zanthoxylum schinifolium KT321318.1, Phellodendron amurense NC_035551.1, Leitneria floridana NC_030482.1.
